# Genetics without genes: application of genetic algorithms in medicine

**DOI:** 10.3325/cmj.2019.60.177

**Published:** 2019-04

**Authors:** Branimir K. Hackenberger

**Affiliations:** Department of Biology, Josip Juraj Strossmayer University, Osijek, Croatia *hackenberger@biologija.unios.hr*

A rector of one university has recently asked me: “I understand the connection between mathematics and physics, but what may be the connection between mathematics and biology?” Indeed, it is a common understanding that there is a relationship between mathematics and physics. After all, many universities offer a double major in mathematics and physics. Why is this so? Is mathematics really so distant from other natural sciences? If not, then why isn't it completely normal to say, for example, a mathematics and biology study program? Why are we separating these two natural sciences? Why are we doing so much to keep biology and biomedical sciences far away from mathematics' love hug? One of the possible answers lies in the dynamics of the development of individual natural sciences, ie, in different timing of their emergence from a set of natural phenomena descriptions. In other words, in the timing of quantification of certain natural sciences. Mathematics is by itself quantitative, whether it is algebra, algebraic topology, fractal geometry, or category theory. Physics became quantitative very early on. In fact, even in antiquity, at the very beginning of the discovery and description of physical phenomena, there was a need for their quantification. Unlike physics, chemistry became quantitative in the 17th century, mostly thanks to the work of Anglo-Irish chemist Robert Boyle. Biology began to quantify a century later. Although some authors consider the work of Fibonacci from the 12th century as the beginning of quantification, it was not until the 18th century, ie, until the works of Daniel Bernoulli, Thomas Malthus, and later Francois Verhulst, that biology as an exact scientific discipline emerged from the collection of descriptions of natural phenomena related to the living world. As Bernoulli and Malthus mainly quantified spreading of disease, they can also be regarded as the initiators of modern epidemiology. However, if someone searched for the interactions between mathematics and biology throughout history, they would find surprisingly many of them. The problems that could not be solved at the level of description drove the development of mathematical tools and theories. Biology has more or less influenced the development of statistical theories, stochastic process theories, dynamic systems theory, nonlinear partial differential and functional equations, topology geometry, etc. On the other hand, mathematical concepts have helped the development of contemporary biology and, consequently, biotechnology and biomedicine. One of the rare mathematical concepts whose name contains a biological component are genetic algorithms (GAs). Similar to how design and creation of many technical inventions (airplane wings, structural elements of buildings, aerodynamic forms, etc) were inspired by nature, GAs were inspired and based on genetic phenomena and natural selection. GAs are inevitable when it comes to contemporary data usage and artificial intelligence, with their main advantage being the ability to solve far more complex problems than those that can be solved by traditional methods. In medicine, the application of GAs is not limited to image processing, but includes very complex use in radiology, oncology, cardiology, endocrinology, gynecology, pediatrics, surgery, pulmonology, infectology, radiotherapy, rehabilitation medicine, orthopedics, neurology and pharmacology – hence in almost every branch of medicine (1). Actually, at least one chapter could be written about the application of GA in each of these areas. GAs are machine-learning and optimization techniques that are used to solve complex search problems. In medical science and engineering, GAs are used to search for huge numbers of possible combinations of parameters in order to find the combination that best suits the realistic or desired endpoint.

**Figure 1:**
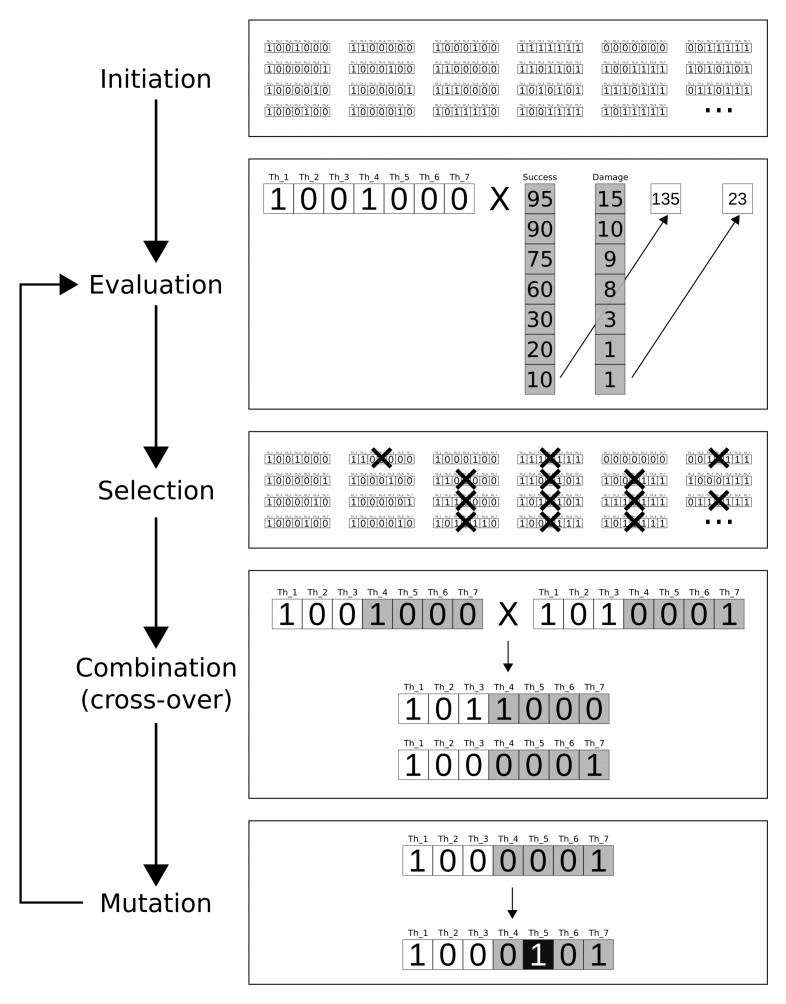
Schematic illustration of the principles of genetic algorithm.

The basic flow of GA consists of five parts (Figure 1). Through the initialization part, a random virtual population of a certain size is created. Population size may vary from several to thousands of individuals. In the next step, the fitness of each individual is determined. Fitness is defined as the proportion in which an individual satisfies the property required by the algorithm. In the third step, this population is improved by keeping only those individuals that best match the required properties. Hence, this step is called a selection. The selection is followed by crossover, in which new individuals are created with the combinations of the existing, ie, survived individuals. In this step, the assumption is to get the individuals that better fit the desired property. The crossover is followed by a mutation step, in which individuals undergo small random changes. After the mutation, the whole sequence is repeated, starting from the evaluation step until the population with the desired properties is obtained.

**Figure 2:**
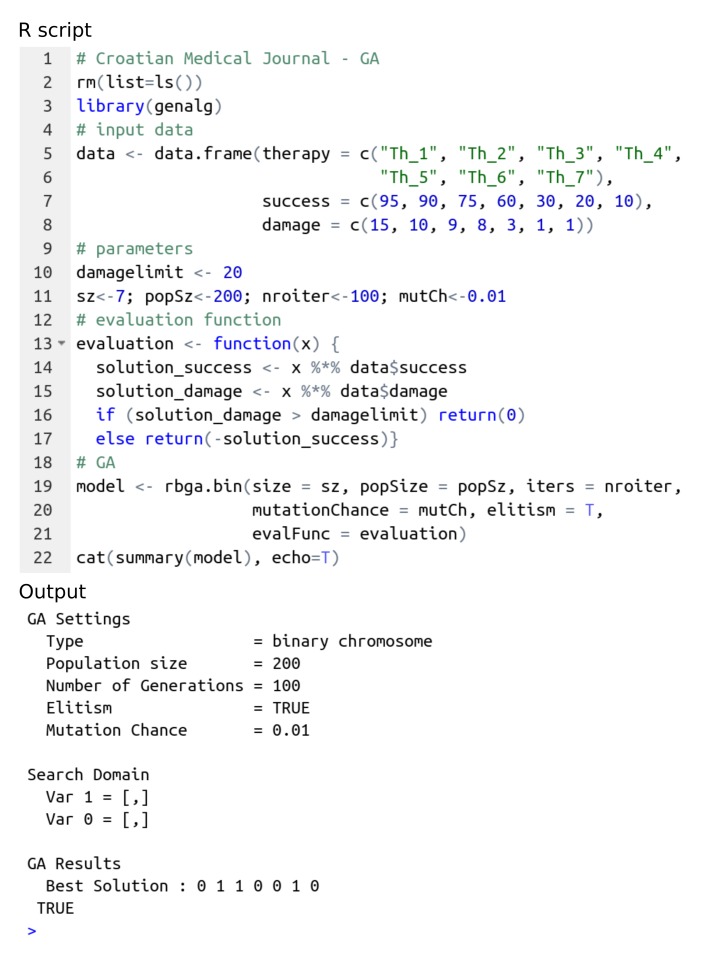
R script and output as an example of usage of optimization of therapy combination.

**Figure 3:**
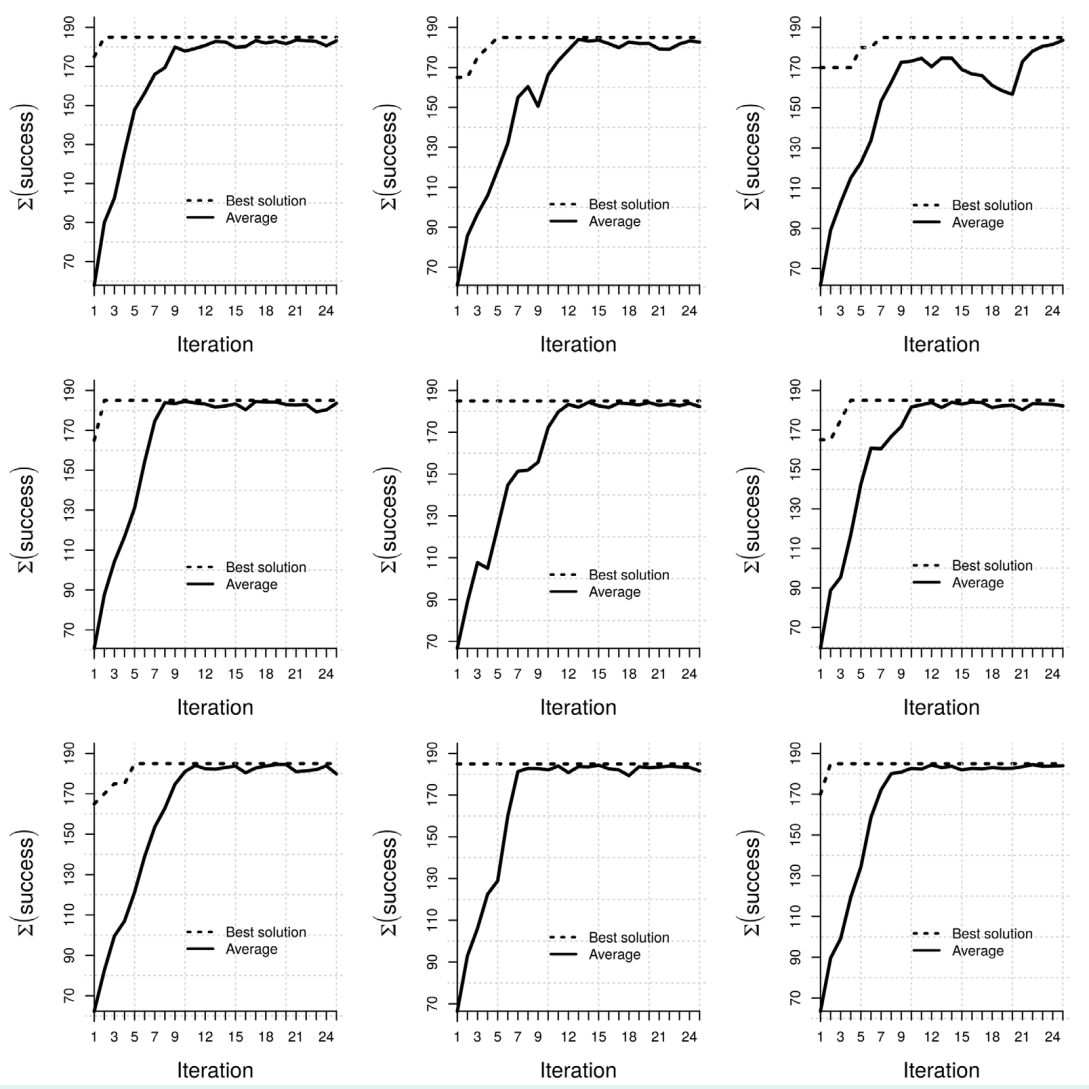
View of the genetic algorithm workflow during the first 25 iterations when solving the same problem.

GAs usage in medicine can be explained using a very simplified case of therapy optimization. Let us suppose that for the treatment of a patient seven different therapies are available that can be combined and that do not interact with each other. Each therapy causes measurable damage to the organism. The chance of success of each therapy and the harm to the organism are shown in Table 1. The question is how many therapies should be applied to achieve the highest likelihood of a successful treatment with the likelihood of harm to the organism not exceeding 20%. The R script that can solve this problem is shown in Figure 2. Since we have seven different therapies, first we create the initial population of “chromosomes” composed of seven “genes.” The GA follows all the steps described until an optimal “genome” is obtained. As the mutation is random, each calculation provides a different output sequence. The final solution is of course equal (Figure 3) – the optimal combination is Th2+Th3+Th6. The described example is a simplified presentation of application of GA for therapy optimization. However, sophisticated GA-based mathematical tools are already widely applied in medicine. Only in the past few years hundreds of scientific and professional articles have been published using some kind of GA. For example, a neural network based on GA (GANN – Genetic Algorithm Neural Network) has in the late nineties been successfully used to predict the outcomes of non-small cell lung cancer surgical treatments (2). GANN systems constructed in this manner can predict far better than the methods based on the usual logistic regression. One of the reasons for GANN’s superiority in such cases is the ability to work with a far greater number of variables. GANN can also be successfully used to interpret the EEG signal. Thus, in 2013, an article describing the use of GA to detect a hypoglycemic condition based on an EEG signal was published (3). GAs can be used in conjunction with other techniques, such as recursive local floating enhancement technique (LFE) (4). Two key steps in GA, combination (crossover) and mutation, ensure that the entire population of each generation will go to the ideal, ie, needed stage. On the other hand, GA cannot improve the individual to its local optimum. LFE greatly enhances the GA’s ability to use the optimal solution for each individual in every generation. In this way, by using such a hybrid GA, a greater number of combinations can be processed simultaneously. An example of the use of such a hybrid GA is the detection of seven protein markers to determine the risk of major adverse cardiac events (5). Another technique is supporting vectoring machines (SVM) algorithm, ie, supervised learning model with related learning algorithms that analyze the data used for regression analysis and classification. GA in conjunction with SVM was used to predict the cardiovascular fetal state (6). This combination of GA and machine learning technique was shown to be superior to an artificial neural network trained for the same purpose (6). GAs can also be used to detect QRS complexes for an automated interpretation of electrocardiograms (7). GAs are also unavoidable for gene selection at microarray gene expression profiling (8). In fact, there is almost no single -omics discipline in which GA does not play a significant role. GA can be used to detect the optimal architecture of artificial neural networks, eg, those that can detect abnormal EEG signals from patients with epilepsy (9). Three hybrid GAs were developed and successfully applied to solve the feature-selection problem for lung transplants with the aim of achieving high classification accuracy for predicting the quality of life for lung transplant patients (10).

**Table 1:** Hypothetical therapies with associated chances of success and damage to the organism

Therapy	Success	Damage
Th_01	95	15
Th_02	90	10
Th_03	75	9
Th_04	60	8
Th_05	30	3
Th_06	20	1
Th_07	10	1

The ways to apply GAs in medicine are constantly being developed, especially in the areas where the use of GA has largely settled, as in the case of ECG interpretation (11). Further development and application of artificial intelligence, both in medical science and in medical practice, will make the development of GA algorithms even more important in terms of creating new, faster, and more powerful GA hybrids with other mathematical and computational tools. In addition, many computer languages (eg, Python) or program environments (R, Matlab) already have default packages or modules with ready-made solutions that allow relatively easy use of GA for data processing as well as creating their own predictive models.
